# Indirect comparison of intravenous vs. subcutaneous C1-inhibitor placebo-controlled trials for routine prevention of hereditary angioedema attacks

**DOI:** 10.1186/s13223-019-0328-3

**Published:** 2019-03-07

**Authors:** Jonathan A. Bernstein, Huamin Henry Li, Timothy J. Craig, Michael E. Manning, John-Philip Lawo, Thomas Machnig, Girishanthy Krishnarajah, Moshe Fridman

**Affiliations:** 1 University of Cincinnati College of Medicine and Bernstein Clinical Research Center, 231 Albert Sabin Way ML #563, Cincinnati, OH 45267-0563 USA; 2 Institute for Asthma and Allergy, Chevy Chase, MD USA; 3 Penn State University College of Medicine, Hershey, PA USA; 4 Medical Research of Arizona, Scottsdale, AZ USA; 5 CSL Behring, Marburg, Germany; 6 CSL Behring, King of Prussia, PA USA; 7 AMF Consulting, Inc., Los Angeles, CA USA

**Keywords:** C1-inhibitor deficiency, C1-INH protein, Crossover studies, Self-administration, Subcutaneous, Intravenous, C1-inhibitor replacement therapy, HAEGARDA, Cinryze

## Abstract

**Introduction:**

For prophylaxis of hereditary angioedema (HAE) attacks, replacement therapy with human C1-inhibitor (C1-INH) treatment is approved and available as intravenous [C1-INH(IV)] (Cinryze^®^) and subcutaneous [C1-INH(SC)] HAEGARDA^®^ preparations. In the absence of a head-to-head comparative study of the two treatment modalities, an indirect comparison of data from 2 independent but similar clinical trials was undertaken.

**Methods:**

Two similar randomized, double-blind, placebo-controlled, crossover studies were identified which evaluated either C1-INH(SC) (COMPACT; NCT01912456; 16 weeks) or C1-INH(IV) (CHANGE; NCT01005888; 14 weeks) vs. placebo (on-demand treatment only) for routine prevention of HAE attacks. Individual patient data from each trial were used to conduct an indirect comparison of treatment effects. Attack reductions (absolute and percent of mean/median number of monthly HAE attacks reduction over placebo) were compared between the two C1-INH formulations at approved/recommended doses: C1-INH(SC) 60 IU/kg twice weekly (n = 45) and 1000 U of C1-INH(IV) twice weekly (n = 22). Point estimates were adjusted using mixed and quantile regression models that controlled for study design.

**Results:**

The absolute mean monthly numbers of HAE attack reductions were 3.6 (95% CI 2.9, 4.2) for C1-INH(SC) 60 IU/kg vs. placebo and 2.3 (1.4, 3.3) for C1-INH(IV) vs. placebo; between-product difference, 1.3 (0.1, 2.4; *P *= 0.034). The mean percent reduction in monthly attack rate was significantly greater with C1-INH(SC) as compared with C1-INH(IV) (84% vs. 51%; *P *< 0.001). The percentages of subjects experiencing ≥ 50%, ≥ 70%, and ≥ 90% reductions in monthly HAE attack rates versus placebo were significantly higher with C1-INH(SC) 60 IU/kg as compared to C1-INH(IV) 1000 U (≥ 50% reduction: 91% vs. 50%, odds ratio [OR] = 10.33, *P *= 0.003; ≥ 70% reduction: 84% vs. 46%, OR = 6.19, *P* = 0.005; ≥ 90% reduction: 57% vs. 18%, OR = 6.04, *P* = 0.007).

**Conclusion:**

Within the limitations of an indirect study comparison, this analysis suggests greater attack reduction with twice-weekly C1-INH(SC) 60 IU/kg as compared to twice-weekly C1-INH(IV) 1000 U for the routine prevention of HAE attacks.

## Introduction

Hereditary angioedema (HAE) is a rare and potentially fatal autosomal dominant disorder with various subtypes. Deficiency or dysfunction of the C1 esterase protein (C1-INH) are the underlying pathophysiology in the most common variants of HAE, known as C1-INH-HAE types I and II, respectively. Hereditary angioedema has a significant adverse impact on functioning and quality of life (QoL), including high levels of anxiety, elevated rates of depression, and disruptions in productivity [1–6].

Recent guidelines recommend long-term prophylaxis to reduce the frequency and severity of attacks in patients who suffer frequent HAE attacks, whose condition is not adequately controlled with on-demand therapy, or who have other disease burden factors [7–10]. Oral attenuated androgens are not completely effective, and their usefulness can be limited by safety/tolerability issues, particularly in certain patient populations (e.g., women, children) and with long-term use [8, 9]. Thus, the most recent World Allergy Organization (WAO) guidelines [10] recommend C1-INH replacement as first-line treatment for long-term prophylaxis. C1-INH replacement addresses the fundamental underlying deficiency in patients with C1-INH-HAE, restoring the physiologic presence and activity of the missing protein, including regulation of bradykinin pathways. C1-INH for prophylaxis has been available as an intravenous formulation since 2008 in the US, and since 2011 in Europe (Cinryze^®^; Shire ViroPharma, Lexington, MA, USA). Suboptimal disease control and breakthrough attacks are common with intravenous C1-INH (C1-INH[IV]) at recommended doses [4, 11, 12]. According to the results of a recent survey, about 20% of patients using C1-INH(IV) prophylaxis experienced breakthrough HAE attacks once a month, and more than 10% experienced attacks two to three times per week [4].

A subcutaneous, highly concentrated, volume-reduced C1-INH concentrate (C1-INH[SC]; HAEGARDA^®^; CSL Behring, Marburg, Germany) was approved by the US FDA in 2017 for routine prevention of HAE attacks in adolescents and adults [13] based on a phase 3, placebo-controlled crossover study, the Clinical Studies for Optimal Management of Preventing Angioedema with low-volume subcutaneous C1-inhibitor Replacement Therapy (COMPACT) trial (NCT01912456). The COMPACT study demonstrated safety and efficacy of C1-INH(SC) for preventing HAE attacks [14] and improved quality of life in patients [5] as compared to on-demand treatment only.

The comparative clinical efficacy of the two approved treatment options, intravenous and subcutaneous replacement therapy with C1-INH, has not been evaluated in a head-to-head trial. Such information will be of considerable interest to clinicians who are involved in treatment decisions for their patients with HAE. Indirect comparisons are recognized as a viable option contributing to the total body of evidence for evaluating medical interventions [15]. In the absence of head-to-head data, an indirect comparison of individual patient data from similar, independently conducted studies was undertaken as an alternate approach to compare the efficacy of twice weekly C1-INH(SC) and C1-INH(IV) at approved doses for prevention of HAE attacks in patients with type I or II C1-INH-HAE.

## Methods

### Systematic literature review for comparative data

A search was performed to identify appropriate comparison data, looking for similarly-designed, placebo-controlled studies with C1-INH(IV) that could be compared to the COMPACT data. The search was performed in PubMed using the terms “C1-inhibitor” and “placebo” and was limited to “human” and “clinical trial.” Potential comparative studies included placebo-controlled trials of C1-INH(IV) used as routine/long-term prophylaxis in HAE that enrolled patients with baseline attack frequency similar to that required for COMPACT (i.e., ≥ 2 attacks per month). Study endpoints must have included HAE attack rates while using C1-INH(IV) and placebo.

One placebo-controlled study of C1-INH(IV) for routine HAE prophylaxis was identified that met the comparative criteria (CHANGE trial; Cinryze^®^ LEVP 2005-1/Part B, NCT01005888) [11, 16]. The CHANGE trial had a similar crossover design as COMPACT as well as the inclusion criterion of attack history of ≥ 2 attacks/month. Monthly attack frequency was a study endpoint. Thus, this study qualified as a comparator.

### Study design and patients

**Fig. 1:**
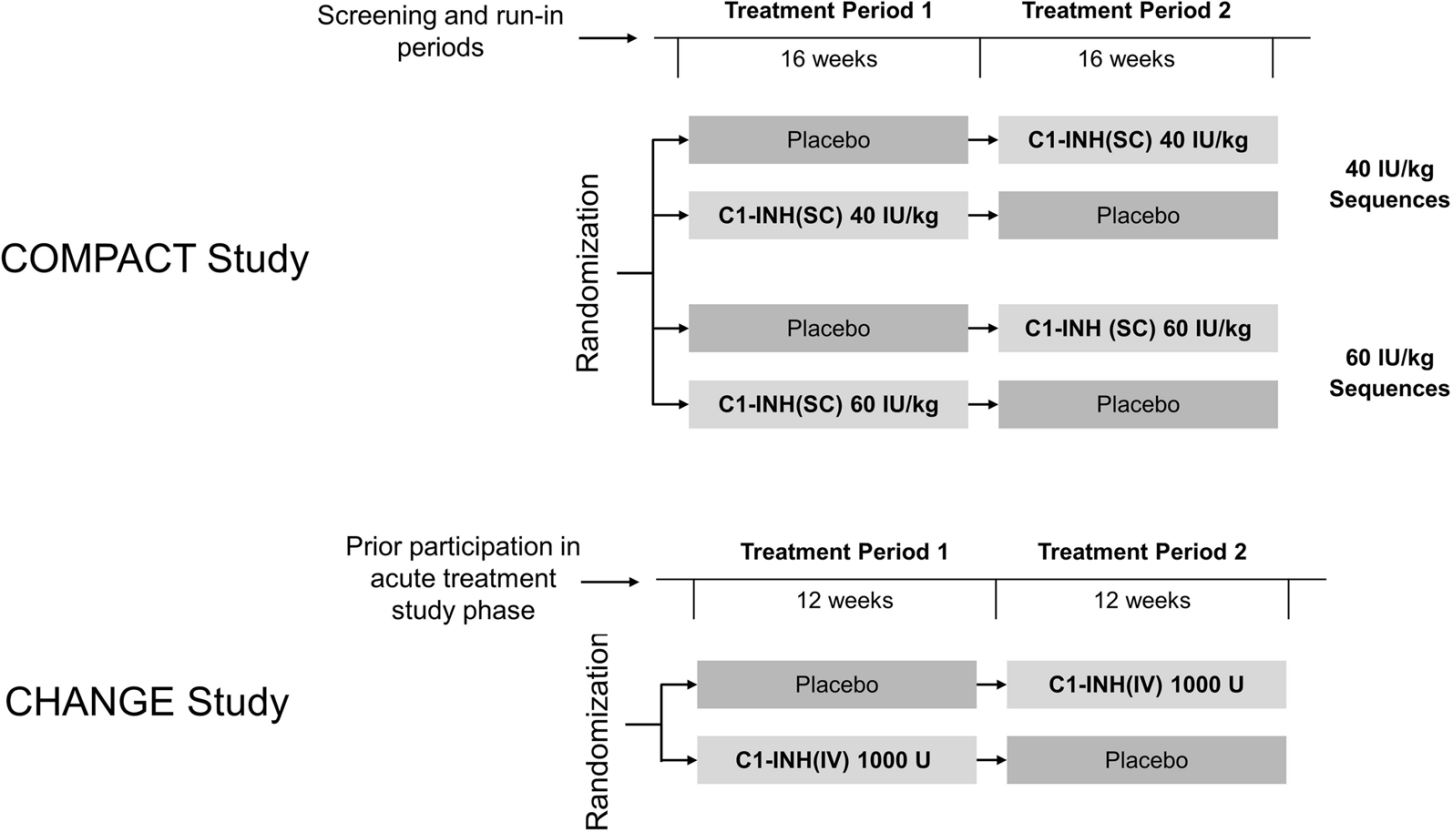
Designs of the COMPACT and CHANGE studies. Study medications were given twice weekly in both studies. *IU* international units, *IV* intravenous, *SC* subcutaneous, *U* units

Individual patient data were available for both trials. For the CHANGE trial, the authors accessed data from the Cinryze^®^ Biologics License Application [16]. The key study design characteristics of both trials are presented in Table 1 and Fig. 1. Of note, both trials had similar inclusion criteria, including requirement for a laboratory-confirmed diagnosis of C1-INH-HAE and a history of at least 2 HAE attacks per month to be eligible to participate. Both studies were crossover designs in which all patients underwent treatment with active drug and placebo in sequential fashion.

**Table 1:** Features of COMPACT [14] and CHANGE [11, 17] studies

	COMPACT	CHANGE
C1-INH formulation	Subcutaneous	Intravenous
Study phase	III	III
Study design	Twice-weekly, dosed by body-weight, C1-INH(SC), 40 or 60 IU/kg for 16 weeks, preceded or followed by placebo for 16 weeks	Twice-weekly, fixed dose, C1-INH(IV) 1000 U for 12 weeks, preceded or followed by placebo for 12 weeks
Inclusion Criteria	Age ≥ 12 years C1-INH-HAE type I/II confirmed by central laboratory ≥ 4 HAE attacks over consecutive 2 months within 3 months before screening Stable oral HAE prophylaxis regimen permitted^a^	Age ≥ 6 years C1-INH-HAE confirmed by laboratory analysis Frequent HAE attacks ≥ 2 per month Normal C1q level
Exclusion Criteria	History of arterial/venous thrombosis requiring anticoagulant therapy or current prothrombotic risk History of poor response to C1-INH therapy for the management of HAE Incurable malignancies C1-INH(IV) routine HAE prophylaxis within 3 months of screening^a^ Pregnant, nursing, or plan to become pregnant during the study Alcohol, drug, or medication abuse within 1 year of screening Known or suspected hypersensitivity to the investigational product Participated in another interventional clinical study within 30 days of screening Unable to have HAE adequately managed with on-demand treatment	Any clinically significant medical condition, e.g., renal failure, that would interfere with participation Presence of anti-C1-INH autoantibody B cell malignancy Participation in a C1 esterase inhibitor trial, or received blood/blood product in the past 90 days Pregnancy or lactation Narcotic addiction History of allergic reaction to C1-INH or other blood products Participation in any other investigational drug study within the past 30 days Low C1q level

*HAE* hereditary angioedema, *IU* international units, *IV* intravenous, *SC* subcutaneous, *U* units

^a^Amendment implemented during the study

### Endpoints

The current analysis included the absolute reduction in the mean and median number of monthly HAE attacks versus placebo treatment, the percentage reduction in the time-normalized number of HAE attacks vs. placebo treatment, and the proportions of subjects with ≥ 50%, ≥ 70%, and ≥ 90% reductions in HAE attacks vs. placebo treatment.

### Statistical analysis

An indirect comparison of FDA-approved dosages for fixed-dosing of C1-INH(IV) (1000 U twice a week) and C1-INH(SC) 60 IU/kg relative to placebo were conducted using the standard methods for indirect comparisons as described by Bucher et al. [18]. This approach to compare treatments across trials with a single common comparator (in this case, the placebo arms) preserves the strength of randomization by comparing the relative effect estimate of treatment A vs. placebo with that of treatment B vs. placebo. To account for study designs, mixed models were used including fixed factors for treatment, period, sequence (as indicator for placebo in the first period) and random effect of subject in the study where possible (see Appendix). Some models were modified due to convergence issues. Models were then used to obtain treatment effect estimates (i.e., C1-INH[SC] versus placebo and C1-INH[IV] versus placebo) and its differences (i.e., [placebo_C1-INH(SC)–C1-INH(SC)]–[placebo_C1-INH(IV)–C1-INH(SC)]). Therefore, the data generated by this analysis can differ from the results published for the individual studies.

For the treatment comparison of reduction over placebo in the number of attacks (normalized for the subject’s treatment duration) a least-squares mean difference was estimated with 95% confidence intervals and P-values. Median % reduction in number and % of monthly HAE attacks over placebo were derived from quantile regression models with 95% confidence intervals and P-values. The percentages of subjects experiencing 50%, 70%, and 90% reduction rates over placebo were compared using odds ratio (OR) derived from mixed logistic regression models.

In the COMPACT study, 5 patients had missing data for 1 of the 2 treatment periods; the 5 missing treatment periods were not included in models with “period” as the unit of analysis, and the 5 patients were excluded from models with “patient” as the unit of analysis. For the CHANGE study, all patient and period data were available and included in the analysis.

Because the CHANGE study had a higher percentage of females, a sensitivity analysis of COMPACT data for females only (n = 32) was performed comparing to all subjects of the CHANGE study (only 2 of 22 patients were male) as the actual gender was not available in the CHANGE patient-specific dataset. This sensitivity analysis was intended to provide an assessment of whether differences in the gender distribution between the studies may have impacted outcomes. Statistical analyses were done using SAS version 9.4 (SAS Institute Inc., Cary, NC). As this was an explorative indirect comparison between 2 studies, P-values were not adjusted for multiplicity. The alpha level was set to 0.05.

## Results

### Subjects

Demographic data for 45 subjects from the COMPACT study were compared to the data of 22 subjects from the CHANGE study (Table 2). The mean subject age was similar between the COMPACT study (37 years) and CHANGE study (38 years) subjects. A majority of subjects in both study populations were female, although more so in the CHANGE study (90.9%) as compared to the COMPACT study (71.1%). Mean body weight in the COMPACT study was slightly higher than in the CHANGE study (80 vs. 73 kg; likely due to the gender difference). In both populations, a majority of subjects had HAE type I.

**Table 2:** Demographics of the study populations

	COMPACT study C1-INH(SC) 60 IU/kg (N = 45)	CHANGE study C1-INH(IV) 1000 U (N = 22)
Age, y		
Mean (SD)	36.8 (14.9)	38.1 (17.2)
Median (range)	35.0 (14, 72)	38.5 (9, 73)
Gender, n (%)		
Female	32 (71.1)	20 (90.9)
Male	13 (28.9)	2 (9.1)
Race, n (%)		
White	44 (97.8)	21 (95.5)
Black/African-American	1 (2.2)	1 (4.5)
Asian	0	0
Other	0	0
Weight, kg		
Mean (SD)	80.2 (24.6)	73.4 (18.9)
Median (range)	78.0 (43.0, 156.8)	69.3 (37.6, 113.9)
HAE type, n (%)		
Type 1	37 (82.2)	18 (81.8)
Type 2	8 (17.8)	4 (18.2)

*HAE* hereditary angioedema, *IU* international units, *IV* intravenous, *SC* subcutaneous, *SD* standard deviation, *U* units, *y* years

### Attack reduction

The absolute mean monthly reduction in number of HAE attacks versus placebo was significantly greater among subjects treated with C1-INH(SC) in the COMPACT study as compared to that in subjects treated with C1-INH(IV) 1000 U in the CHANGE study (3.6 vs. 2.3 attacks; *P *= 0.034) (Table 3).

**Table 3:** Efficacy endpoints with C1-INH(SC) and C1-INH(IV) in the placebo-controlled COMPACT and CHANGE studies, respectively

	COMPACT study^a^ C1-INH(SC) 60 IU/kg (N = 40)^c^	CHANGE study^b^ C1-INH(IV) 1000 U (N = 22)	Adjusted difference^d^	P-value
Monthly reduction in number of attacks vs. placebo				
Least-squares mean (95% CI)	3.6 (2.9, 4.2)	2.3 (1.4, 3.3)	1.3 (0.1, 2.4)	0.034
Adjusted median (95% CI)	3.1 (2.3, 4.4)	2.2 (1.9, 3.6)	1.2 (− 0.9, 3.3)	0.251
Attack rate reduction vs. placebo (%)				
Least-squares mean (95% CI)	84.0 (75.6, 92.4)	50.8 (30.4, 71.3)	32.8 (14.4, 51.2)	<0.001
Adjusted median (95% CI)	95.1 (86.4, 100.0)	53.1 (30.7, 88.3)	42.7 (11.6, 73.7)	0.008

Adjusted data for the crossover study design variables—means adjusted for period and sequence and medians for sequence within treatment (period not included for convergence reasons); may vary slightly from previously reported unadjusted data

*CI* confidence interval, *IU* international units, *IV* intravenous, *SC* subcutaneous, *U* units

^a^Observation period of 14 weeks

^b^Observation period of 12 weeks

^c^Five of the 45 patients in COMPACT had missing attack values (3 during the placebo period and 2 during the C1-INH[SC] 60 IU/kg period) and were excluded from analyses

^d^Model-based estimates for differences in outcomes

Mean and median percent reductions in HAE attack rate versus placebo were significantly (*P *≤ 0.008) greater among subjects treated with C1-INH(SC) 60 IU/kg as compared to C1-INH(IV) 1000 U (Table 3). The mean values for monthly attack percent reduction rates were 84% (C1-INH[SC] 60 IU/kg) vs. 51% (C1-INH[IV] 1000 U); *P *< 0.001. Median values for monthly attack percent reduction rates were 95% with C1-INH(SC) 60 IU/kg and 53% with C1-INH(IV), corresponding to a 43% improvement with C1-INH(SC) 60 IU/kg (*P *= 0.008).

**Fig. 2:**
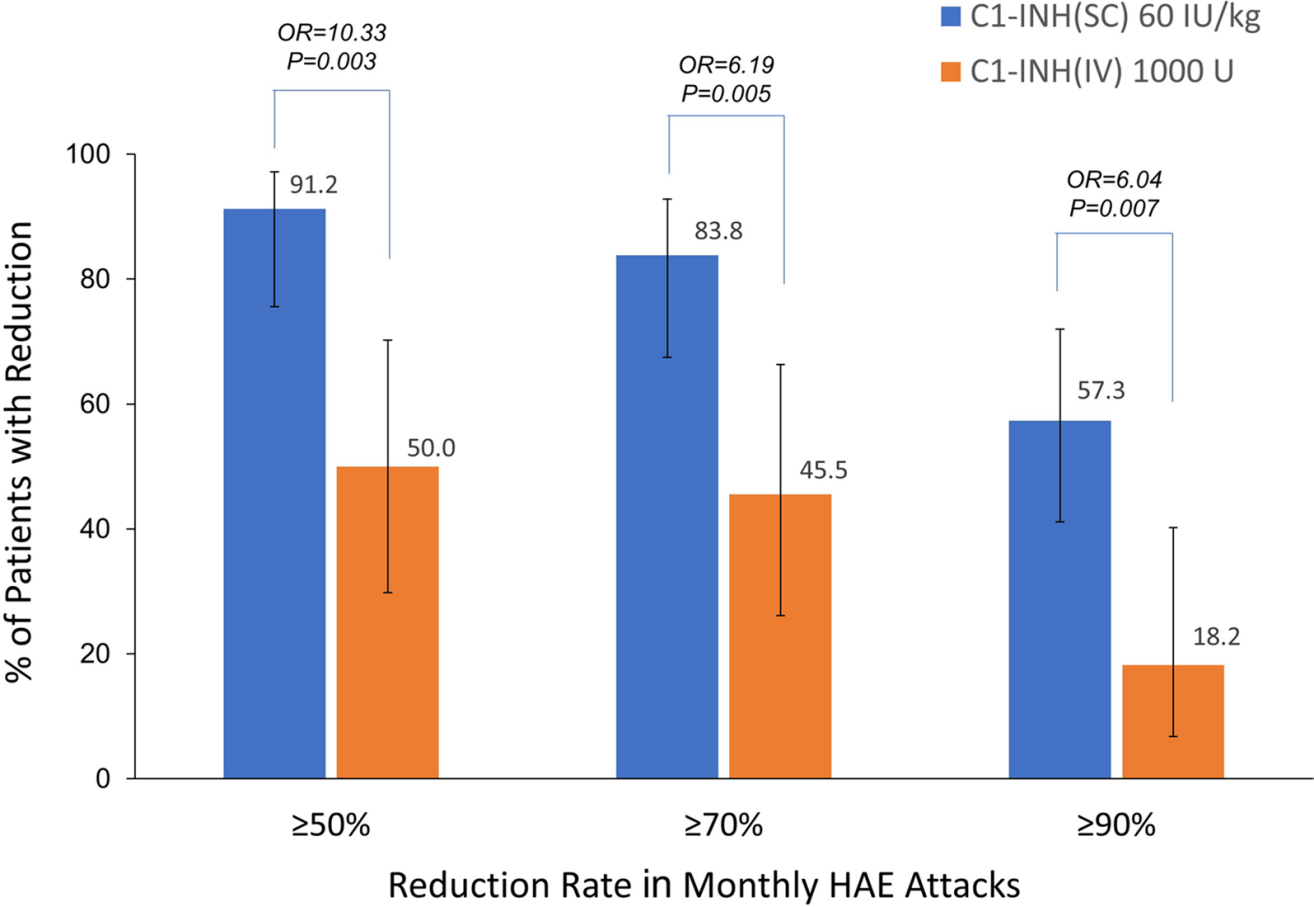
Percentages of subjects with ≥ 50%, ≥ 70%, and ≥ 90% reductions in the number of monthly HAE attacks on active treatment relative to placebo and adjusted odds ratios. Adjusted data for the crossover study design variables sequence within treatment (period not included for convergence reasons); may vary slightly from previously reported unadjusted data. Five of the 45 patients in COMPACT had missing attack values (3 during the placebo period and 2 during the C1-INH[SC] 60 IU/kg period) and were excluded from analyses. Bars represent 95% CIs. Products were administered twice weekly. The observation period was 14 weeks for the COMPACT study (C1-INH[SC] data) and 12 weeks for the CHANGE study (C1-INH[IV] data). *CI* confidence interval, *HAE* hereditary angioedema, *IU* international units, *IV* intravenous, *SC* subcutaneous, *U* units

The percentages of subjects experiencing ≥ 50%, ≥ 70%, and ≥ 90% reductions in monthly HAE attack rates versus placebo were significantly higher with C1-INH(SC) 60 IU/kg as compared to C1-INH(IV) 1000 U (≥ 50% reduction: 91% vs. 50%, OR = 10.33, *P *= 0.003; ≥ 70% reduction: 84% vs. 46%, OR = 6.19, *P* = 0.005; ≥ 90% reduction: 57% vs. 18%, OR = 6.04, *P* = 0.007) (Fig. 2).

In a sensitivity analysis comparing COMPACT data for females only (n = 32) to all subjects of the CHANGE study (91% females), similar results were observed, with significantly greater percentages of subjects experiencing ≥ 50%, ≥ 70%, and ≥ 90% reductions in HAE attack rates versus placebo among those receiving C1-INH(SC) 60 IU/kg versus C1-INH(IV) 1000 U (≥ 50% reduction, 89% vs. 50%, OR = 8.37, *P *= 0.009; ≥ 70% reduction, 82% vs. 45%, OR = 5.30, *P* = 0.015; ≥ 90% reduction, 51% vs. 18%, OR = 4.66, *P* = 0.028).

## Discussion

This is, to the best of our knowledge, the first and only indirect comparison of data from two independent studies investigating the preventive effect of two approved, long-term C1-INH replacement therapies for the routine prevention of HAE attacks. Within the limitations of an indirect study comparison, these results suggest a statistically significant and clinically meaningful difference between twice-weekly C1-INH(SC) 60 IU/kg and twice-weekly C1-INH(IV) 1000 U. Greater reductions in both mean and median HAE attack rates were observed with C1-INH(SC) as compared to C1-INH(IV). Notably, 91% of subjects treated with C1-INH(SC) 60 IU/kg achieved a ≥ 50% reduction in attack rate, and more than half (57%) attained a ≥ 90% reduction in attack rate, versus 50% and 18% of those using C1-INH(IV) 1000 U, respectively. Based on these results, patients would be expected to experience approximately one less attack per month on average using the SC dosing regimen compared to the IV regimen.

In the absence of a head-to-head trial, this analysis was conducted using an indirect comparison of patient-level data from two clinical trials with similar study designs and patient populations. Treatment comparisons using indirect evidence from independently conducted studies are not as scientifically vigorous as data analyzed as part of a direct, comparative study; nonetheless, such analyses are considered valid alternatives in the absence of direct comparative data [15]. Both source studies were high quality and met the similarity assumption requiring two factors important to the internal validity of indirect comparisons [15]; both studies used crossover designs, and the comparable nature of the patient populations is supported by the finding that the time-normalized attack rates during placebo treatment were similar (i.e., about 4 attacks per month, i.e., frequent attackers, in both studies).

When extrapolating findings from this indirect clinical trial comparison of two approved dosing regimens of C1-INH to the real-world setting, several confounding factors have to be taken into consideration. In real-world practice, physicians can individualize C1-INH dosing regimens and titrate as necessary for maximum effectiveness as the clinical situation may require. While the CHANGE study used a fixed dose of C1-INH(IV) 1000 U, the official prescribing information for C1-INH(IV) allows for consideration of doses up to 2500 U (not to exceed 100 U/kg) every 3 or 4 days depending on individual patient response [19]. This was based on findings from a dose-escalation study in which increasing twice-weekly doses of C1-INH(IV) from 1000 U to as much as 2500 U (in 500 U increments) further reduced the attack rate in patients not well controlled on 1000 U; although most patients experienced some attack reduction benefits with higher doses, 4 of 12 patients (33%) who were escalated to the maximum dose of 2500 U were still considered failures [20].

With C1-INH(SC), long-term and real-world data are limited as of yet. Recent findings from an open-label study in patients exposed > 24 months indicated that the preventive effect of treatment increased over time with > 80% of patients becoming attack-free after the end of the final 6-month observation period [21]. Thus, a definitive assessment of the comparative long-term effectiveness of C1-INH(IV) and C1-INH(SC) remains speculative and warrants further investigation.

This study has several limitations. First, even though baseline patient characteristics and general designs of the individual studies were similar, the sample sizes were relatively small, the trial populations were comprised of distinct sets of patients, treatment periods and study conduct were unique to each study, and there may have been potentially unmeasured confounders which may have influenced treatment outcomes; such issues are less likely to affect treatment comparisons within a head-to-head randomized trial. Yet, this indirect comparison also has several strengths. Each study included a placebo arm as a common comparator. For both studies individual patient data were available and due to the crossover study design applied in both trials, each patient served as its own control, thus reducing variability of attack reduction endpoints.

The relative risk of experiencing an HAE attack has been inversely correlated with increasing C1-INH functional activity levels [14, 22]. While pharmacokinetic (PK) data could not be compared between the COMPACT and CHANGE studies due to unavailability of PK data from the CHANGE study, a recent PK simulation comparing different modes of administration of C1-INH showed higher minimum trough functional C1-inhibitor activity levels with C1-INH(SC) 60 IU/kg administration (~ 48%) compared with IV administration (different formulation than used in the CHANGE study) at doses of 1000 U (~ 30%) or 2500 U (~ 38%) [23]. Higher trough values have been shown to be associated with a reduced risk of experiencing an attack based on PK and pharmacodynamics (PD) modeling [14, 22]. Thus, our observations from this indirect study comparison are biologically plausible and consistent with what would have been predicted from PK/PD simulations.

Efficacy issues aside, the option of SC administration of C1-INH can overcome barriers associated with IV administration, including emotional/psychological barriers and physical obstacles such as venous access. A recent online survey of adults with HAE found that 30% of peripheral vein users of C1-INH(IV) were somewhat dissatisfied or not at all satisfied with their treatment, and a majority reported some difficulty in finding usable veins or administering the infusion [12]. Additionally, the need for frequent IV administration sometimes requires the use of central venous access devices which are associated with various medical risks (e.g., thrombosis, infection) and functional complications (e.g., blockage) [12, 24–26].

## Conclusions

In summary, within the limitations of this indirect study comparison, we conclude that subcutaneously administered C1-INH replacement therapy at the approved dosage of 60 IU/kg provides a greater and clinically meaningful additional preventive effect against HAE attacks as compared to C1-INH(IV) at the approved dosage of 1000 U twice weekly. Our findings are consistent with prior evidence regarding the exposure–response relationship of C1-INH replacement and attack risk.

## Appendix

See Table 4.

**Table 4:** Model specifications and reported statistics by study endpoint

Endpoint	Model	Reported statistics
Mean difference in monthly attacks	SAS PROC MIXED: fixed effects: treatment, period, sequence, random effect: subject within treatment	Point estimates for treatment effect over placebo and 95% CI; difference of treatment effects between C1-INH(SC) and C1-INH(IV), 95% CI and P-value
Median difference in monthly attacks	SAS PROC QUANTREG: fixed effects: treatment, sequence within treatment	
% reduction in monthly attacks	SAS PROC MIXED: fixed effects: treatment, period, sequence within treatment	
Median % reduction in monthly attacks	SAS PROC QUANTREG: fixed effects: treatment, sequence within treatment	
Number of patients with ≥ × % reduction	SAS PROC GLIMMIX: fixed effects: treatment, sequence within treatment	Percentage of patients; odds ratio C1-INH(SC) over C1-INH(IV) and P-value

*CI* confidence interval

## Abbreviations

C1-INH: C1-inhibitor
COMPACT trial: Clinical Study for Optimal Management of Preventing Angioedema with Low-Volume Subcutaneous C1-inhibitor Replacement Therapy trial
HAE: hereditary angioedema
IV: intravenous
PD: pharmacodynamic
PK: pharmacokinetic
QoL: quality of life
SC: subcutaneous
WAO: World Allergy Organization
